# Supplementary material for: Investigating Fibroblast-Induced Collagen Gel Contraction Using a Dynamic Microscale Platform
Source: Front Bioeng Biotechnol. 2019 Aug 14;7:196. doi: 10.3389/fbioe.2019.00196 (PMC6702460; doi:10.3389/fbioe.2019.00196)
Supplement: Supplementary file 1 [file Data_Sheet_1.PDF]

Investigating Fibroblast-Induced Collagen Gel Contraction Using a Dynamic Microscale Platform

Supplementary Material

Tianzi Zhang[1†], John H. Day[1†], Xiaojing Su[1], Arthur G. Guadarrama[2], Nathan K. Sandbo[2], Stephane Esnault[2], Loren C. Denlinger[2], Erwin Berthier[1], and Ashleigh B. Theberge[1,3*]

[1]Department of Chemistry, University of Washington, Seattle, Washington, USA

[2]Department of Medicine, University of Wisconsin School of Medicine and Public Health, Madison, Wisconsin, USA

[3]Department of Urology, University of Washington School of Medicine, Seattle, Washington, USA

[†] **Equal Contribution**

*** Correspondence:**
Corresponding Author
abt1@uw.edu

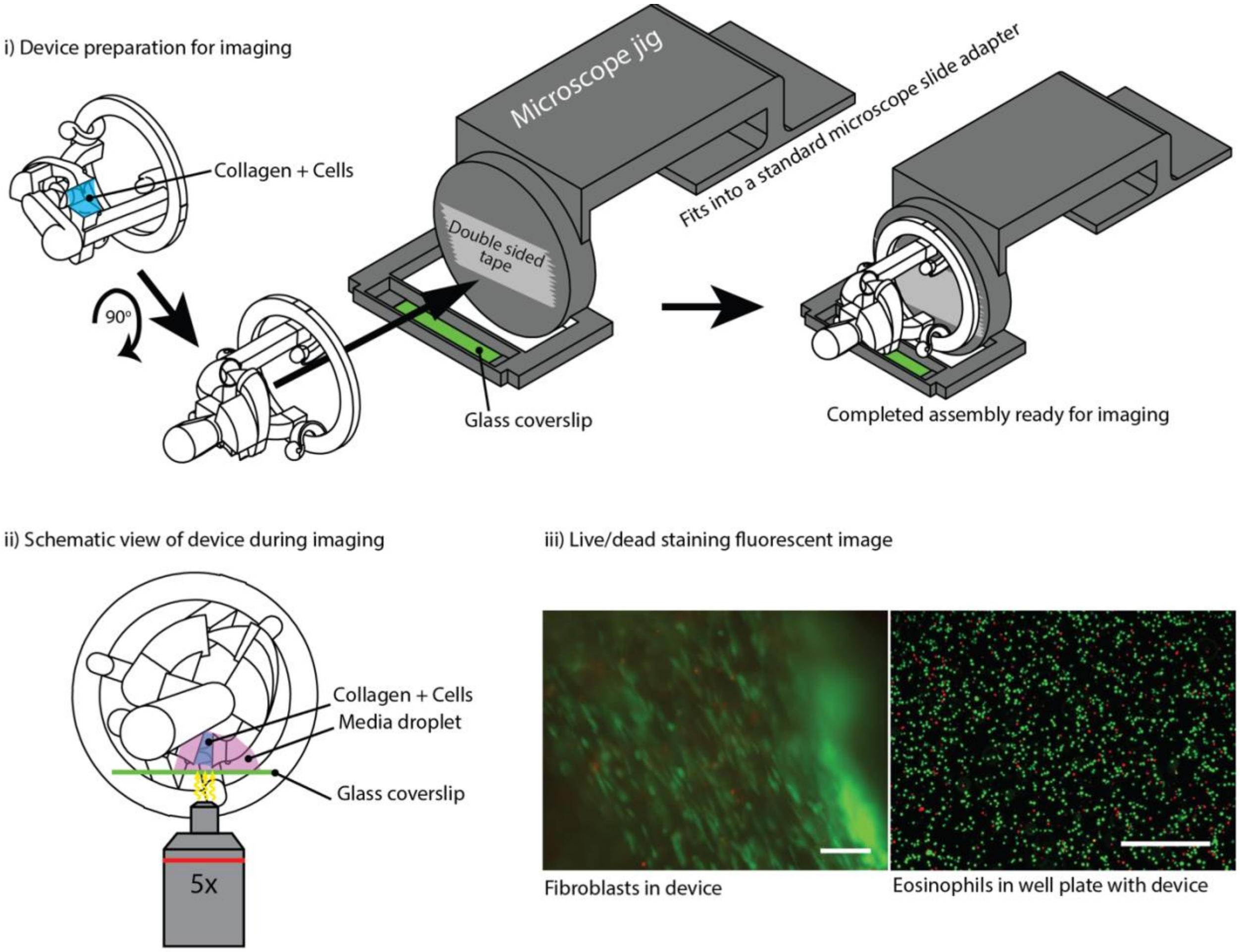

Supplementary Figure 1: Imaging in CGC device. i) Imaging in device requires a microscope jig, which positions the collagen bridge directly above a glass slide. ii) Schematic view of imaging in device. Microscope jig is omitted for simplicity. iii) Fluorescence images of fibroblasts in collagen and eosinophils in the well plate (green: live, red: dead, scale bars: 100 μm).

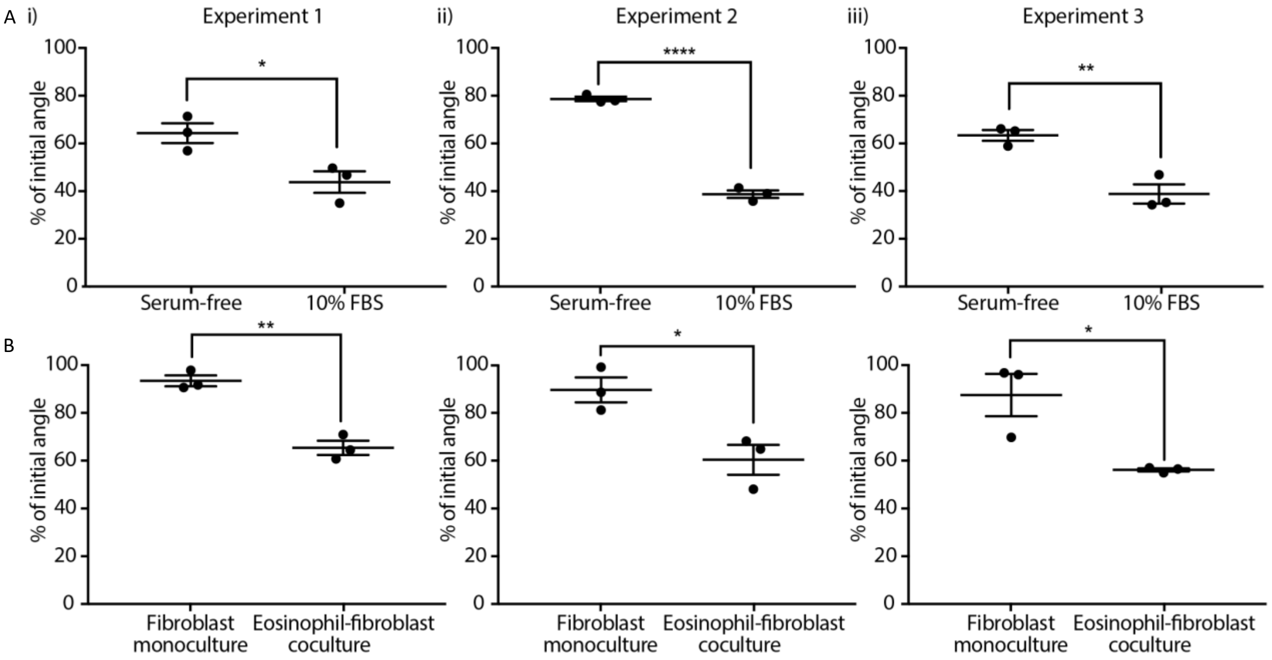

Supplementary Figure 2: Complete set of data collected across three independent experiments; each data point plotted is from a replicate device within an independent experiment. These are the complete data sets data corresponding to (A) Figure 2B and (B) Figure 3B. Error bars: SEM of three device replicates; p-value indicates significantly different values according to a two-tailed unpaired Student's t-test (*p≤ 0.05, **p ≤ 0.01, ****p≤ 0.0001).

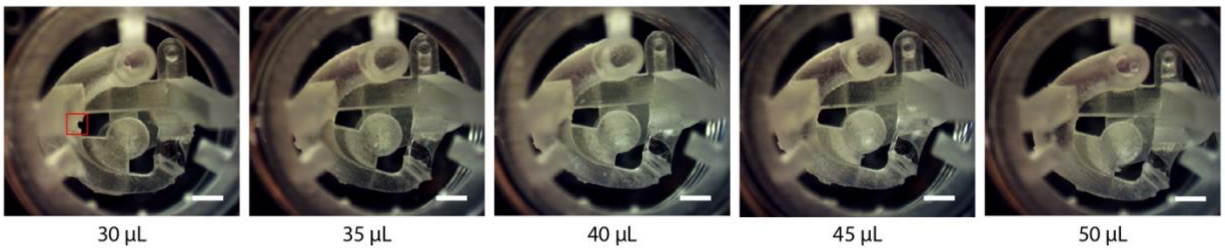

Supplementary Figure 3: Determination of the volume of media required when loading the retraction tube such that the free-swinging arm is pulled back and the collagen droplet extends into a collagen bridge (step ii) and iii) of Figure 1). Each image is the representative of four devices for each loading volume. The loading was successful with a volume of 35, 40, 45, and 50 µL of media. With a loading volume of 30 µL, there was not enough media to bring the tail of the swing arm completely back into the retraction tube, resulting in a small gap (red box). Scale bars: 2 mm.

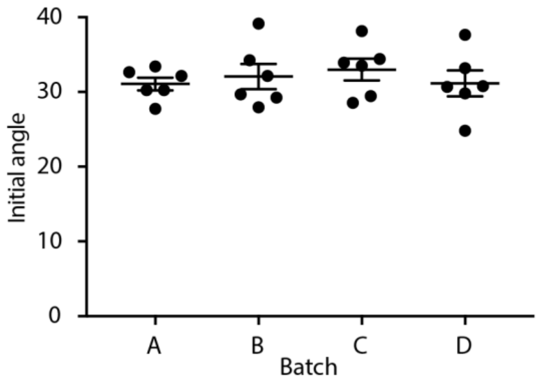

Supplementary Figure 4: The initial angle of six devices (each plotted as a separate data point) containing collagen with no cells from four different batches (3D printed on different days). Error bars represent SEM.

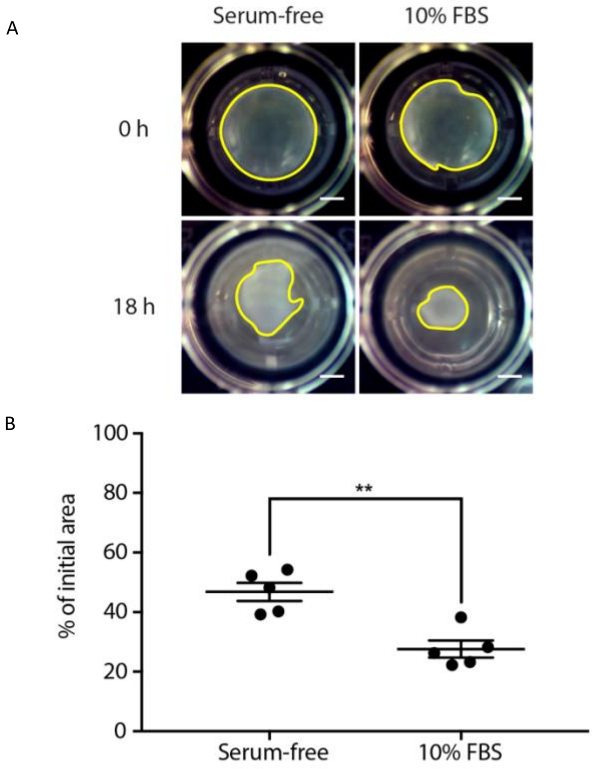

Supplementary Figure 5: HFL-1 induced collagen gel contraction in a 96 well plate, using the standard macroscale collagen gel contraction assay. (A) The gel contours are traced in yellow, showing gel disc distortion. (B) Each data point represents the percentage of initial gel area of each replicate from an independent experiment. Error bars: SEM of 5 replicates; p-value indicates significantly different values according to a two-tailed unpaired Student's t-test ($^{**}p \leq 0.01$). Scale bars: 1 mm.

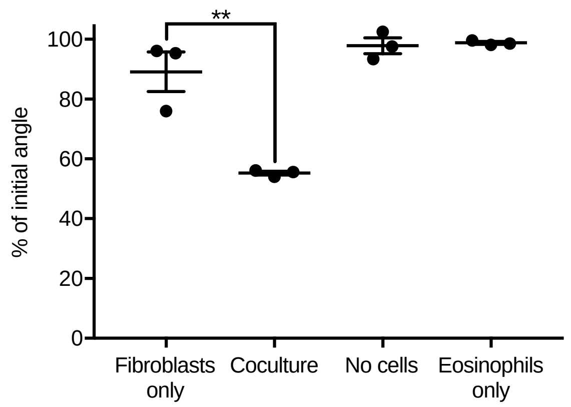

Supplementary Figure 6: HFL-1 mediated collagen contraction in serum-free media under four different culture conditions. “No cells” and “Eosinophils only” conditions are used as negative controls. Each data point represents a single device from the same experiment. Error bars: SEM; ** indicates significantly different values according to a two-tailed unpaired Student's t-test ($p \leq 0.01$); only the “Fibroblasts only” and “Coculture” conditions were compared.

Approximate determination of surface tension forces in CGC device.

The force provided by the surface tension of media in the retraction tube is equal and opposite to the force applied by the surface tension of the collagen bridge. This force can be calculated using the following equation:

$$F = PA = \gamma(\frac{1}{r_1} + \frac{1}{r_2})A$$

Where P is the Laplace pressure associated with the air-liquid interface, A is the surface area of that interface, $\gamma$is the surface tension of the liquid, and r_1 and r_2 are the characteristic radii of curvature that describe the interface. If we assume that the collagen bridge is approximately cylindrical, and that its surface tension is equal to the surface tension of pure water, then we can calculate the surface tension force applied by the collagen bridge:

$$\gamma = 72 \times 10^{-3} \, \frac{m\mathrm{N}}{\mathrm{m}}$$

$$r_1 = 1.014 \times 10^{-3} \text{ m}$$

$$r_2 \cong \infty$$

$$A \cong \left(2 \times 10^{-3} \left(\frac{3.172 + 1.477}{2}\right) (\pi \times 1.014 \times 10^{-3})\right)$$

Note that the surface area of the collagen bridge is approximated as the area of two trapezoids with width of parallel edges given by measurements 2 and 3 from Figure S7, and height given by half the circumference of the cylindrical bridge.

$$F = PA = 72 \times 10^{-3} \left(\frac{1}{1.014 \times 10^{-3}} + \frac{1}{\infty}\right) \left(2 \times 10^{-3} \left(\frac{3.172 + 1.477}{2}\right) (\pi \times 1.014 \times 10^{-3})\right)$$

Using these assumptions, and by measuring the diameter of the collagen bridge in FIJI image processing software, we find that the collagen bridge applies a force of approximately 1 mN, and thus the media in the retraction tube also applies 1 mN in opposition. However, the surface tension force applied by the media in the retraction tube is nullified when the device is submerged in media at the beginning of an experiment, thus enabling the device to move dynamically based on forces exerted by the cells on the collagen.

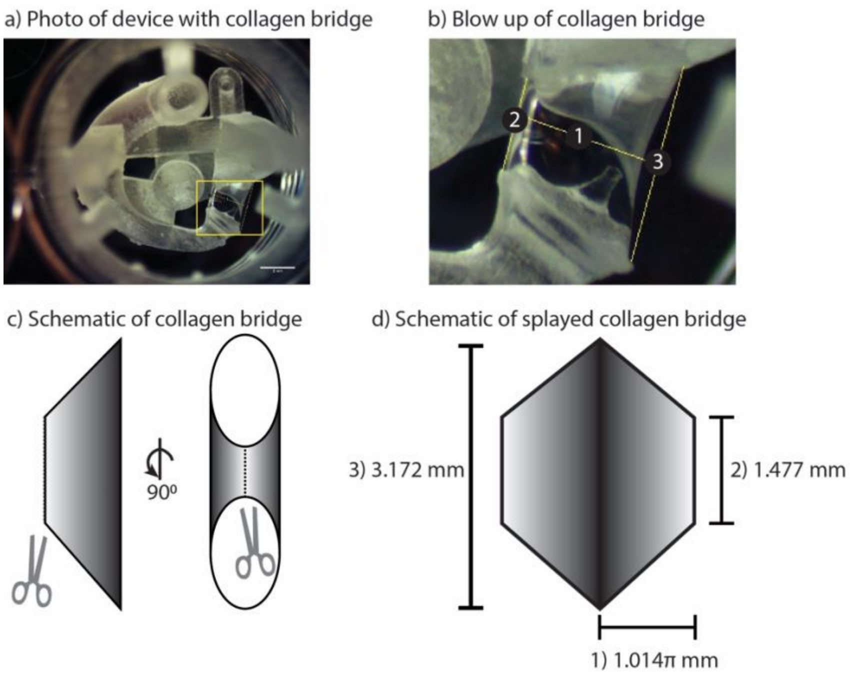

Supplementary Figure 7: Photo used to make retraction force approximation and corresponding schematic representation of surface area approximation. a) Photo of device used for approximation with b) inset of collagen bridge. Yellow lines indicate where measurements were taken. c) Schematic of 3D shape used to approximate collagen bridge (cylinder with angled end caps). d) Schematic of approximate 2D (splayed out) surface geometry of collagen bridge. Measurements from b) were used to calculate the area of this shape. Scale bar: 2 mm.
